# Bacille Calmette-Guérin (BCG) spondylitis with adjacent mycotic aortic aneurysm after intravesical BCG therapy: a case report and literature review

**DOI:** 10.1186/s12879-018-3205-7

**Published:** 2018-06-28

**Authors:** Takuya Kusakabe, Kenji Endo, Itaru Nakamura, Hidekazu Suzuki, Hirosuke Nishimura, Shinji Fukushima, Kengo Yamamoto

**Affiliations:** 1 Department of Orthopedic Surgery, Tokyo Medical University, 6-7-1 Nishi-shinjuku, Shinjuku-ku, Tokyo, 160-0023 Japan; 2 Department of Infection Prevention and Control, Tokyo Medical University, 6-7-1 Nishi-shinjuku, Shinjuku-ku, Tokyo, 160-0023 Japan

**Keywords:** Intravesical BCG therapy, BCG spondylitis, Mycotic aortic aneurysm, T-SPOT, Interferon-gamma release assay (IGRA), Case report

## Abstract

**Background:**

Although intravesical bacille Calmette-Guérin (BCG) therapy is accepted as an effective treatment for bladder cancer, serious complications may occur in rare cases. To date, only 4 cases have been reported in which the patient developed a combination of mycotic aortic aneurysm and BCG spondylitis. Accurate diagnosis of BCG spondylitis is important because it is an iatrogenic disease, and its treatment is different from usual tuberculous spondylitis. However, distinguishing BCG spondylitis from usual tuberculous spondylitis is very difficult and takes a long time. In this study, we were able to suspect BCG spondylitis at an early stage from the result of the interferon-gamma release assay (IGRA).

**Case presentation:**

We encountered a case of BCG spondylitis with adjacent mycotic aortic aneurysm after intravesical BCG therapy in a 76-year-old man*.* We performed a 2-stage operation to obtain spine stabilization and replace the aneurysm with a synthetic graft. We started multidrug therapy with antituberculosis medication, excluding pyrazinamide, because the patient’s history of BCG therapy, negative IGRA, and positive of tuberculosis-polymerase chain reaction (Tb-PCR) suggested that the pathogenic bacteria of the spondylitis was BCG. Eventually the bacterial strain was identified as BCG by PCR-based genomic deletion analysis.

**Conclusions:**

BCG infection should be considered in patients who have been treated with BCG therapy, even if the treatment was performed several months to several years previously. In the case of a patient with a history of BCG therapy, a positive Tb-PCR result and negative IGRA result probably suggest BCG infections, if the possibility of false-negative IGRA result can be excluded.

## Background

Since the first use of intravesical bacille Calmette-Guérin (BCG) therapy by Morales et al. in 1976, it has been shown to be an effective treatment for the prevention and treatment of superficial bladder carcinoma, including carcinoma in situ [1]. When BCG is instilled into the bladder, it provokes an inflammatory response with mononuclear cell infiltration and class-II major histocompatibility complex expression in malignant cells. The malignant cells then become a target for lymphokine-activated killer cells and BCG antigen-presenting cells [2].

Although BCG therapy for bladder cancer is accepted as an effective treatment, serious complications, such as bone and joint infections, including in the spine, may occur in rare cases. Accurate diagnosis of BCG spondylitis is important because it is an iatrogenic disease, which is likely to be misdiagnosed as a metastatic bone tumor or vertebral fracture, and its treatment is different from usual tuberculous spondylitis. Here we report a very rare case of late-onset BCG spondylitis with an adjacent mycotic aortic aneurysm after septic shock, which appeared to be a consequence of BCG therapy.

## Case presentation

A 76-year-old man was referred to our hospital for having lower back pain for 5 months, which was suspected to be L2/3 spondylitis on magnetic resonance imaging (MRI). He had previously undergone cervical laminoplasty for ossification of the posterior longitudinal ligament and diffuse idiopathic skeletal hyperostosis 19 years earlier. He had a history of hypertension and diabetes. Although a history of BCG vaccination was unknown, he had no previous history of tuberculosis infection. He had also undergone transurethral resection of a bladder tumor (TUR-Bt) and had been treated with intravesical mitomycin C (MMC) for the bladder cancer 1.5 years earlier. Four months later, he again underwent TUR-Bt, received intravesical MMC, and started BCG therapy for the recurrence of bladder cancer. After the sixth course of intravesical BCG therapy, he was aware of systemic weakness and loss of appetite, and was unable to walk. He appeared to have septic shock and therefore was treated in the intensive care unit. In spite of systemic analyses, the source of his infection and the causative bacteria could not be identified. He was subsequently diagnosed with hypercytokinemia caused by BCG therapy. He showed clinical improvement without the administration of antituberculosis drugs, and was discharged 9 months before he came to our hospital.

Except for a temperature of 37.2 °C, his vital signs were within normal limits. Although there were no motor and sensory disturbances in the legs, he was unable to walk owing to lower back pain. Physical examination demonstrated vertebral tenderness at the L2/3 level. Laboratory analysis demonstrated a normal white blood cell (WBC) count of 6300 /μL, a high erythrocyte sedimentation rate (ESR) of 53 mm/h, and a high C-reactive protein (CRP) level of 2.7 mg/dL. There were no other abnormal laboratory findings regarding anemic changes, kidney function, or liver function. The patient did not receive tuberculin skin testing.

**Fig. 1:**
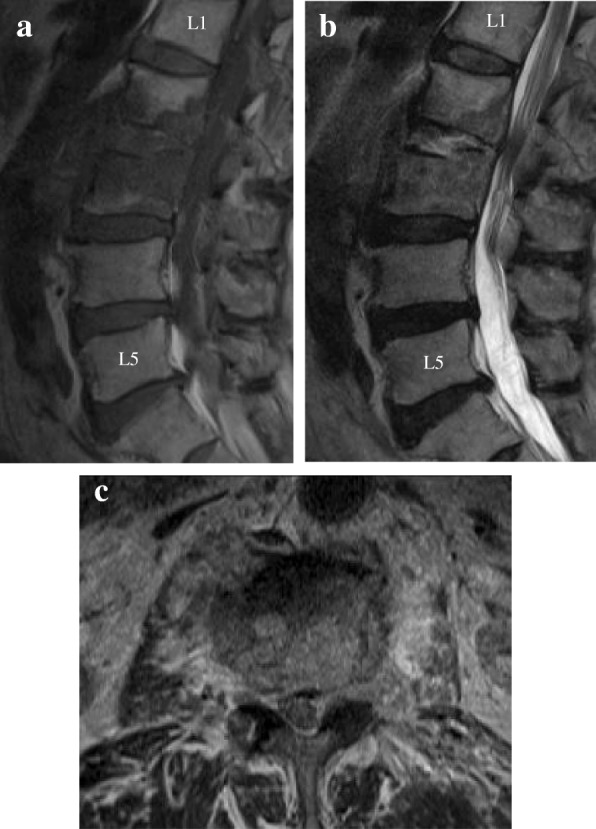
Magnetic resonance images of our patient. **a.** Sagittal T1-weighted images displaying a decreased signal in the L2/3 disc and the vertebral bodies. **b.** Sagittal T2-weighted images displaying an increased signal in the L2/3 disc and fluid collection in the anterior part of the vertebral bodies. **c.** Axial T2-weighted images displaying an increased signal around the posterior portion of the vertebral bodies, which extended into the left epidural space and reached the peripheral muscle tissue and the area near the aorta

There were no notable findings on electrocardiogram or chest X-ray. X-ray of the lumbar spine displayed collapsed endplates of L2/3. Sagittal T1-weighted MRI displayed a decreased signal in the L2/3 disc and the vertebral bodies (Fig. 1a). Sagittal T2-weighted MRI displayed an increased signal in the L2/3 disc and fluid collection in the anterior part of the vertebral bodies (Fig. 1b). Axial T2-weighted MRI displayed an increased signal around the posterior area of the vertebral bodies, which extended into the left epidural space and reached the peripheral muscle tissue and the area near the aorta (Fig. 1c).

**Fig. 2:**
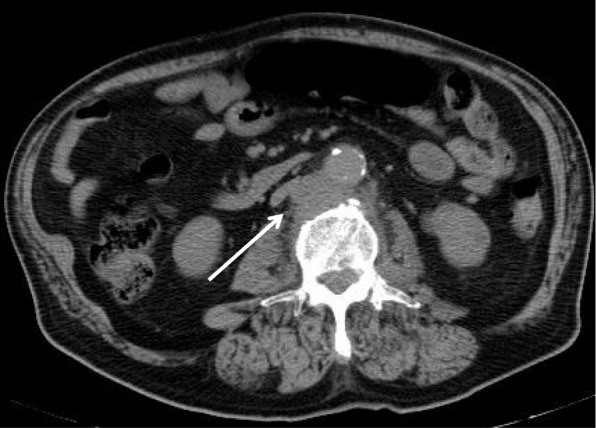
Plain abdominal computed tomography scan of our patient. The mycotic abdominal aortic aneurysm is shown by an arrow

**Fig. 3:**
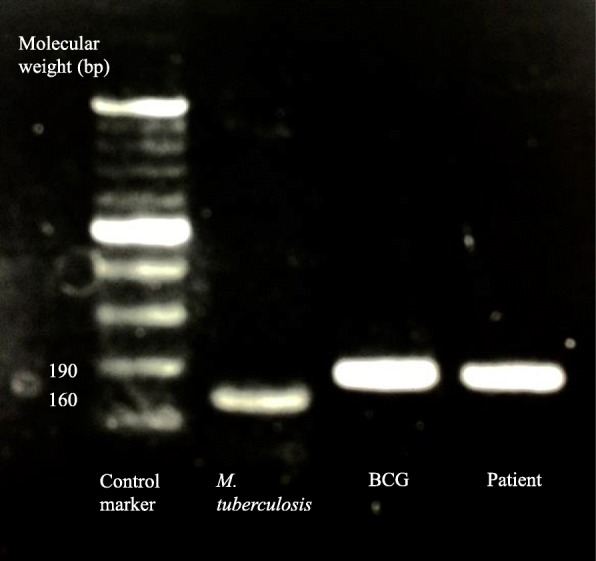
Polymerase chain reaction-based genomic deletion analysis. A clinical isolate sample from our patient was identified as bacille Calmette-Guérin with a deletion in region of difference 1. *M. tuberculosis*: *Mycobacterium tuberculosis*; BCG: bacille Calmette-Guérin; Patient: our patient in this case

On the second day of hospitalization, the patient underwent an L2/3 disc biopsy, but the general bacteria culture was negative, and the smear was negative for Ziehl-Neelsen staining. The patient’s blood culture was negative, and the result of T-SPOT.TB® (T-SPOT), which is a type of interferon-gamma release assay (IGRA), were also negative; the number of spots for both 6-kDa early secretory antigenic target (ESAT-6) and 10-kDa culture filtrate antigen (CFP-10) was 5 or less. The causative bacteria remained unidentified and therefore he was started on empirical therapy with intravenous ceftriaxone. On the fifth day of hospitalization, a plain computed tomography scan, which was performed for systemic examination, showed soft tissue development in the adjacent abdominal aorta at the L3 level (Fig. 2), which was suspected to be an infectious aortic aneurysm. A 2-stage operation was planned for the spondylitis with adjacent infectious aortic aneurysm, to prevent rupture of the infectious aortic aneurysm, obtain spine stabilization, drain the abscess and make a diagnosis. On the tenth day in hospital, because the risk of rupture was considered to be low, the patient underwent an L2/3 laminectomy followed by posterior fixation using percutaneous pedicle screws at T12, L1, L4, and L5 as the first stage. Specimen cultures of the lumbar vertebrae, yellow ligament, necrotic tissue, etc., were negative for general bacteria, specimen smears were also negative for Ziehl-Neelsen staining, and there were no pathological findings of caseating granuloma or necrosis. On the fiftieth day of hospitalization, because the infection had been controlled but the inflammatory response was sustained, he underwent replacement of the aneurysm with a synthetic graft by vascular surgeons, and lesion curettage and L2/3 anterior interbody fusion by iliac bone transplantation as the second stage. Spinal surgery was performed in the transabdominal approach owing to the risk of rupture. On pathological analyses, the L2/3 intervertebral disc, vertebral bone, and tissue surrounding the vertebral bone and aorta showed caseating granuloma and necrosis with multinucleated giant cells and epithelioid cells upon hematoxylin-eosin staining, and positive bacilli upon Ziehl-Neelsen staining. The tuberculosis-polymerase chain reaction (Tb-PCR) result of the tissue was also positive, using COBAS® TaqMan® MTB Test, which is a real-time PCR system targeting the 16S rRNA gene region of *Mycobacterium tuberculosis* complex DNA. Owing to the patient’s history of BCG therapy, negative T-SPOT, pathological findings, and positive Tb-PCR, the pathogenic bacteria of the spondylitis was considered to be BCG. We then started multidrug therapy with antituberculosis drugs, including isoniazid (INH), rifampin (RFP), and ethambutol (EB), because BCG is typically resistant to pyrazinamide (PZA). PCR-based genomic deletion analysis was performed using the specimens to distinguish BCG from the other *M. tuberculosis* complexes. Specifically, multiplex PCR was performed utilizing region of difference 1 (RD1), which is present in the DNA of other *M. tuberculosis* complexes but is deleted in the DNA of BCG [3]. Primers ET1, ET2 and ET3 bind and amplify a 190-bp region in BCG, whereas a 160-bp region is amplified in the other *M. tuberculosis* complexes, as observed by electrophoresis on an agarose gel. A clinical isolate sample from our patient was identified as BCG with a deletion in RD1 (Fig. 3). Specimen cultures from the first-stage and second-stage operation were later identified as members of the *M. tuberculosis* complex using a mycobacteria growth indicator tube. After therapeutic intervention, the patient’s WBC count, ESR level, and CRP level were improved and MRI displayed no signs of active infection in the spine, epidural space, peripheral muscle tissue, or aorta. On the ninetieth day after hospitalization, the patient was discharged from our hospital and transferred to a different hospital for physical rehabilitation.

## Discussion

Although BCG therapy can cause adverse effects, such as cystitis (> 90% of adverse effects), fever (2.9%), hematuria (1%), prostatitis (0.9%), and arthralgia or arthritis (0.5%), it is generally considered to be safe [3]. However, rare cases of serious complications, such as interstitial pneumonitis, military tuberculosis, sepsis, infectious aortic aneurysm, iliopsoas abscess, osteomyelitis, and spondylitis have been reported [4–6].

To date, only 22 cases of BCG spondylitis after intravesical BCG therapy have been reported in the English-language literature, and only 4 of these cases had a combination of mycotic aortic aneurysm and BCG spondylitis (Table 1).

**Table 1:** Published cases of BCG spondylitis after BCG therapy (modified and supplemented from Obaid et al. [1] and Dąbrowska et al. [22])

Reference	Age	Time from BCG treatment to onset	Area of disease	Mycotic aortic aneurysm	Antituberculosis drugs used	Surgical intervention	Prognosis
Katz [23]	67	1.3 years	L4–5	No	INH + RFP + EB	Yes	No long-term follow-up
Fishman [24]	90	1.3 years	T11–12	No	INH + RFP + EB	Yes	Not specified
Civen [25]	81	7 months	T12-L1	No	INH + RFP	Yes	Complete recovery
Sugita [5]	71	2 months	T7	No	INH + RFP + SM	Yes	Not specified
Morgan [26]	77	0.5 months	T11-L1	No	INH + RFP + EB	Yes	Functional after 1 year
Rozenblit [9]	76	5.7 years	L4	Yes	INH + RFP + EB + CPFX	No	Asymptomatic at 8 months
Aljada [27]	79	2.5 years	L3	No	INH + RFP	Yes	Leg weakness remaining after 1 year
Abu-Nader [4]	76	7 years	T6–7	No	INH + RFP + EB	No	Symptoms improved
Dahl [28]	69	1 years	L3–4	Yes	INH + RFP	Yes	Complete recovery
Nikaido [29]	86	1.8 years	T12-L1	No	INH + RFP + EB	No	Complete recovery
Mavrogenis [30]	79	12 years	L3–4	No	INH + RFP + EB	Yes	Pain-free at 18 months
Patel [31]	66	5 months	T10–11	No	INH + RFP + EB	No	Symptoms improved at 3-month follow-up
Colebatch [32]	67	5 years	L4–5	No	INH + RFP + EB	No	No long-term follow-up
Josephson [15]	75	6 months	L1–3	No	INH + RFP	No	No long-term follow-up
Obaid [1]	67	11 months	L1–2	No	INH + RFP + EB	Yes	Complete recovery
Santbergen [10]	58	3 years	T8–9	Yes	INH + RFP	Yes	Complete recovery
Samadian [7]	94	5 months	L1–2	Yes	Except PZA	No	No long-term follow-up
Newman [2]	80	3 years	T9–10	No	INH + RFP + EB	Yes	Not specified
Dąbrowska [22]	67	1 month	T10–11	No	INH + RFP + EB	Yes	Motor paralysis remaining
Białecki [33]	66	5 years	T11–12	No	INH + RFP + PZA	Yes	Neurological disorders remaining
Białecki [33]	35	18 months	T5–6	No	INH + RFP + PZA	No	Complete recovery
Miyazaki [34]	82	16 months	T5–6	No	INH + RFP + EB	Yes	Complete recovery

*L* lumbar vertebra, *T* thoracic vertebra, *INH* isoniazid, *RFP* rifampin, *EB* ethambutol, *CPFX* ciprofloxacin, *PZA* pyrazinamide

The average age of these patients was 73.1 (35–94) years and they were all men. Most patients were affected at the lower thoracic and upper lumbar vertebrae with an average of 2.5 years (0.5 months-12 years) after instillation. Based on the treatment for usual tuberculous spondylitis, most patients were treated with antituberculous multidrug therapy for an average of 12 months (9–15 months). However, in all but 2 patients, PZA administration was discontinued at the time of identification of BCG, because BCG is resistant to PZA. Surgical intervention was necessary in 14 patients (64%) who had spinal cord injury, spinal instability and abscess formation. Although motor paralysis remained in a few patients, the conditions of the patients improved or the patients completely recovered and the prognosis was good in most cases after therapeutic intervention.

BCG spondylitis is thought to occur by hematogenous spread through Batson’s plexus, a network of valveless veins that connect the deep pelvic veins to the internal vertebral venous plexuses, which may explain its high incidence in the thoracolumbar spine [1]. Reported risks of complications of BCG therapy that are thought to facilitate its hematogenous spread are the following: transurethral resection of the prostate or bladder biopsy within the previous 2 weeks, trauma to the bladder epithelium from catheterization, deep bladder tumor resection or urethral injury during BCG instillation, hematuria, bladder outlet obstruction, pelvic radiation, and severe cystitis [4]. An immunocompromised state and advanced age were also reported as risks for developing complications. Advanced age is a significant risk factor for complications, with 1 study reporting a rate of 17.6% for those younger than 70 years and 48.6% for those 70 years or older [7]. In our present case, the patient was considered to be at high risk for complications owing to his age (> 70-years old) and his compromised state due to diabetes.

BCG can be detected in bladder biopsy specimens and early morning urine cultures even after more than a year following completion of BCG therapy [8]. In addition, BCG has very low pathogenicity in humans. This may explain why patients are at risk of infection, taking an average of 2.5 years (0.5 months-12 years) after BCG instillation.

To date, at least 20 cases of mycotic aortic aneurysm due to intravesical BCG therapy have been reported in the English-language literature [7, 9, 10]. Although the exact mechanism of mycotic aortic aneurysm due to BCG is not known, 3 possible mechanisms have been described [10]. The first mechanism is hematogenous spread through the vasa vasorum, the second is lymphatic spread through the retroperitoneal lymph nodes, and third is contiguous spread from an infectious focus as a psoas abscess or spondylitis to the aorta [11]. Four cases, including our present case appears to have spread contiguously, because the mycotic aortic aneurysm and BCG spondylitis were at the same level of the spine.

Collaboration with vascular surgeons is important when performing surgery in patients of BCG spondylitis with mycotic aortic aneurysm. It is necessary to consider the activity of the infection, the site of the aortic aneurysm and the risk of its rupture, and the spinal cord injury. In our present case, the patient’s infection had been controlled and the risk of rupture was thought to be low, and therefore, spinal surgery was given priority. Whether the surgery of the infected aortic aneurysm should be performed by in situ reconstruction (ISR) or by extra-anatomical bypass (EAB) remains controversial [12, 13]. One report recommended EAB for cases of infected aortic aneurysm with spondylitis [14]. However, owing to the very low pathogenicity of BCG, ISR combined with antituberculous multidrug therapy appears to be a relatively safe choice [10]. In our present case, because the infection was controlled, ISR was performed.

It is very difficult to diagnose BCG spondylitis, because its symptoms, laboratory findings, imaging results, and pathological findings are similar to usual tuberculous spondylitis. Although tuberculosis infection as well as chronic progressive infections, such as brucellosis, melioidosis, actinomycosis, candidiasis, and histoplasmosis should be suggested as differential diagnoses of BCG infection, it might be difficult to make a diagnosis merely based on clinical symptoms and radiological findings. Therefore, it is very important to consider BCG infection, particularly in patients who have received BCG therapy, even if the treatment was performed years before the present episode. A tuberculin skin test is not very informative for the diagnosis of *M. tuberculosis* complex diseases, because most of the patients are elderly and demonstrate anergy to the skin test [4]. In addition, it is impossible to distinguish BCG infections from usual *M. tuberculosis* infections by a tuberculin reaction. Although BCG can be identified by the culturing of specimens, this takes a few months to obtain results. Although usual PCR and the DNA-probe test can identify the presence of the *M. tuberculosis* complex, they cannot identify the actual species within the *M. tuberculosis* complex. A previous report introduced techniques used to distinguish BCG from other members of the *M. tuberculosis* complex, such as high-performance liquid chromatography and restriction fragment length polymorphism analysis. Unfortunately, these advanced techniques are not available in many clinical laboratories [15]. In our case, the strain was finally identified as BCG by PCR-based genomic deletion analysis. However, we started multidrug therapy with antituberculous drugs (INH, RFP, and EB) at the time of receiving information regarding the patient’s history of BCG therapy, as well as the pathological data, Tb-PCR positivity, and T-SPOT negativity. T-SPOT, which is a type of IGRA, is an immunoassay using ESAT-6 and CFP-10, which are thought to be expressed from RD1 [16–19]. T-SPOT and other IGRAs have high sensitivity and specificity for *M. tuberculosis* infections [20, 21]*.* Therefore, in the case of patients with a history of BCG therapy, Tb-PCR positive and T-SPOT negative results are likely to indicate BCG infections with a deletion of RD1. Furthermore T-SPOT can be readily performed in most general hospitals, because it uses normal blood-collection tubes. As T-SPOT is an immunoassay, it can-not exclude false-negative results, and additional molecular techniques are required for a definitive diagnosis. However, the results of T-SPOT can be clinical clues of BCG infection.

## Conclusions

In conclusion, BCG therapy should be accompanied by appropriate risk assessment, and high risk cases require careful follow-up. BCG infection should be considered in patients who have received BCG therapy, even when the treatment was performed several months to several years previously. In the case of patient with a history of BCG therapy, Tb-PCR positive and IGRA negative results probably suggest BCG infections, if the possibility of false-negative IGRA results can be excluded.

## Abbreviations

BCG: bacille Calmette-Guérin
CFP-10: 10-kDa culture filtrate antigen
CRP: C-reactive protein
EAB: extra-anatomical bypass
EB: ethambutol
ESAT-6: 6-kDa early secretory antigenic target
ESR: erythrocyte sedimentation rate
IGRA: interferon-gamma release assay
INH: isoniazid
ISR: in situ reconstruction
MMC: mitomycin C
MRI: magnetic resonance imaging
PZA: pyrazinamide
RD1: region of difference 1
RFP: rifampin
Tb-PCR: tuberculosis-polymerase chain reaction
TUR-Bt: transurethral resection of a bladder tumor
WBC: white blood cell
